# ANCA-associated vasculitis after hantavirus infection: A case report

**DOI:** 10.1097/MD.0000000000042821

**Published:** 2025-06-13

**Authors:** Seunghyeok Choi, Hyeong Wan Kim, Kyoung Min Kim, Kyung Pyo Kang

**Affiliations:** a Department of Internal Medicine, Jeonbuk National University Hospital, Jeonju, Korea; b Department of Pathology, Jeonbuk National University Medical School, Jeonju, Korea; c Department of Internal Medicine, Research Institute of Clinical Medicine, Jeonbuk National University Medical School, Jeonju, Korea.

**Keywords:** acute kidney injury, antineutrophil cytoplasmic antibody, hantavirus infection, vasculitis

## Abstract

**Rationale:**

Antineutrophil cytoplasmic antibody (ANCA)-associated vasculitis (AAV) is a small and medium-vessel vasculitis that often leads to rapidly progressive glomerulonephritis. Although certain infections have been associated with ANCA formation, their role in triggering AAV is not fully understood.

**Patient concerns:**

A 60-year-old male who tested positive for hantavirus initially presented with fever, cough, and skin rash. Despite treatment with antibiotics for community-acquired pneumonia, his condition worsened, revealing elevated serum creatinine and proteinuria. Subsequent testing identified a significant increase in hantavirus antibody titers and positive anti-myeloperoxidase ANCA antibodies.

**Diagnosis:**

Laboratory tests and subsequent renal biopsy confirmed AAV with hantavirus infection.

**Intervention:**

Oral cyclophosphamide and high-dose glucocorticoids were initiated.

**Outcomes:**

The patient’s renal function deteriorated after immunosuppressive treatment, necessitating maintenance hemodialysis.

**Lessons:**

This is the first reported case of AAV following hantavirus infection, underscoring the need for vigilance in distinguishing AAV from other conditions in patients with viral infections.

## 1. Introduction

Antineutrophil cytoplasmic antibody (ANCA)-associated vasculitis (AAV) is a type of pauci-immune small and medium-vessel vasculitis characterized by a diverse range of nonspecific symptoms, such as fever, malaise, and arthralgia.^[1–4]^ AAV also affects the kidney, presenting as rapidly progressive glomerulonephritis.^[1,2,4]^ The underlying mechanism responsible for triggering AAV remains to be fully elucidated. Various theories have been suggested for the development of ANCA. ANCA formation during viral, bacterial, or fungal infections, has been reported, and infection can induce myeloperoxidase (MPO)-specific AAV.^[5–7]^ The development of MPO-ANCA during infection could lead to the development of vasculitis.^[5]^

Hantavirus infection, commonly transmitted by rodents, manifests as fever, myalgia, hemorrhagic manifestations, and acute kidney injury (AKI).^[8]^ In Asia, the Hantaan and Seoul viruses are the primary causative agents.^[9]^ Hantavirus infections can lead to conditions such as hemorrhagic fever with renal syndrome (HFRS) and hantavirus cardiopulmonary syndrome, which are typically diagnosed via serologic tests for specific antiviral antibodies.^[10]^ There have been occasional reports of hantavirus infections triggering autoimmune responses.^[11,12]^ However, the relationship between vasculitis and hantavirus infection is not well understood.

Here, we present a case of newly diagnosed AAV with MPO-ANCA positivity in a patient with a confirmed hantavirus infection, specifically the Hantaan virus. This case highlights a potential association between hantavirus infection and the initiation of AAV.

## 2. Ethical approval

Ethical approval was obtained from the Jeonbuk National University Hospital Institutional Review Board (CUH 2024-09-012). Written informed consent for publication was obtained from the patient.

## 3. Case

**Figure 1.**
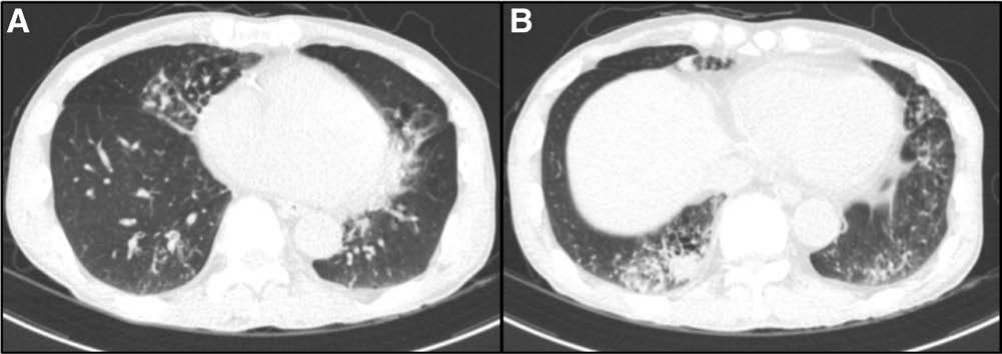
Initial chest computed tomography findings. (A and B) Chest CT showed bilateral and multifocal ground glass opacities and no other evidence of an enlarged lymph node or mass. Emphysema was found in both lung fields.

A 60-year-old male, previously healthy, was admitted to the emergency room with fever, productive sputum, and skin rash. On initial evaluation, we found multifocal ground glass opacities in both lung fields (Fig. 1) and increased C-reactive protein up to 207.66 mg/dL. Serum levels of aspartate aminotransferase/alanine aminotransferase were also elevated to 106/102 IU/L. Hypoalbuminemia (3.4 g/dL) and 1 + proteinuria without hematuria and pyuria were detected on the initial evaluation. We diagnosed community-acquired pneumonia and treated it with intravenous antibiotics, including ceftriaxone and azithromycin. However, we escalated the antibiotics to meropenem because of persistent fever. Despite a change in antibiotics, the fever persisted. To define the lung involvement in other conditions and evaluate the focus of fever, we conducted other laboratory tests, including autoantibody and viral markers. Unexpectedly, the hantavirus IgG antibody was detected at a low titer (1:32), which was inconclusive. Throughout the clinical course, no hypotensive events were observed, therefore, we considered it an atypical clinical course of Korean HFRS. There was a persistent increase in peripheral eosinophilia. However, there was no evidence of Eosinophilic granulomatosis with polyangiitis, such as asthma, sinusitis, or polyneuropathy. We prescribed low-dose prednisolone (15 mg/day) in the suspicion of drug reaction by antibiotics, and the fever subsided.

**Figure 2.**
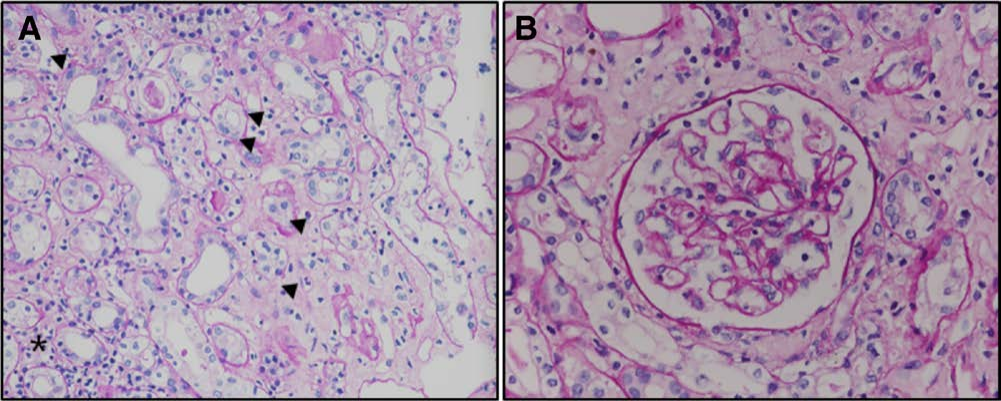
Histopathologic features of the renal biopsy. (A) The tubulointerstitium shows interstitial inflammation (asterisk) and inflammatory cell infiltration within peritubular capillaries (arrowheads) (PAS stain, original magnification × 400). (B) The glomerular basement membrane shows focal and segmental wrinkling, and some glomerular capillary lumina contain lymphocytes. However, crescent formation is not identified (PAS stain, original magnification: ×400). PAS, Periodic acid-Schiff.

**Figure 3.**
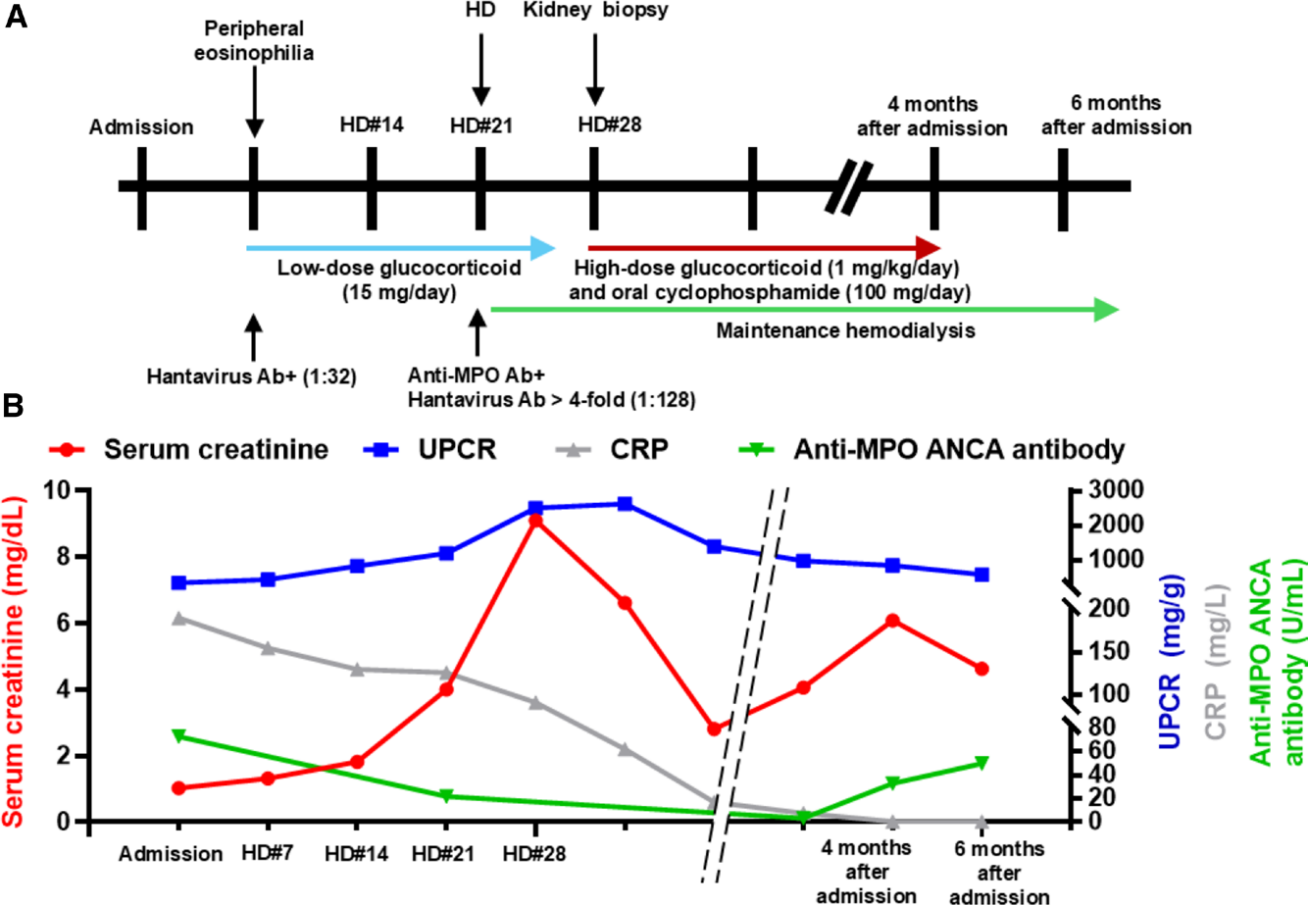
Summary of progression. (A) Diagram depicting brief progression. The patient was admitted to the hospital with a fever and suspicion of pneumonia. Despite the administration of broad-spectrum antibiotics, clinical signs and symptoms did not improve. Although low-dose prednisone treatment relieved fever, AKI occurred. ANCA-associated vasculitis and Korean hemorrhagic fever with renal syndrome were suspected as a cause of AKI and were treated; however, the response was poor, and the patient required permanent dialysis. (B) Changes of serum creatinine (mg/dL; reference range, 0.7~1.2; Red line), urine protein/creatinine ratio (UPCR, mg/g creatinine; Blue line), C-reactive protein (CRP, mg/L; reference range, 0~5.0; Grey line) and anti-MPO ANCA antibody (reference range, <5.0 U/mL, Green line) according to patient progress. AKI, acute kidney injury; ANCA, antineutrophil cytoplasmic antibody; MPO, myeloperoxidase.

After a 1-week course of low-dose prednisolone treatment, his serum creatinine level was slightly elevated to 1.68 mg/dL, compared to the initial results of 1.03 mg/dL, and 1 + proteinuria was still detected. 2 weeks after low-dose prednisolone treatment, he showed coarse breathing sounds with Rhonchi and 2 + pretibial pitting edema on physical examination. Laboratory tests revealed an increased serum creatinine of 4.00 mg/dL and hypoalbuminemia (2.9 g/dL). On urinalysis, hematuria (>50/high power fields) was observed, and the urine protein/creatinine ratio was 833.33 mg/g. Complete blood count showed 24,450/μL of white blood cells, 8.3 g/dL of Hb, and 607,000/μL of platelets. Anti-MPO ANCA antibody was positive at 73.7 U/mL, and other autoantibodies, including antinuclear antibody, anti-double-stranded DNA antibody, and anti-Ro/SSA and La/SSB antibody, were negative. The hantavirus IgG antibody titers increased by >4-fold (1:128) in the follow-up test conducted 3 weeks later, compared to the initial result. We suspected AAV and atypical Korean HFRS. Despite conservative care and low-dose prednisolone (15 mg/day), the patient’s renal function deteriorated, and hemodialysis was initiated. After a few sessions of dialysis, the patient stabilized, and we were able to perform a kidney biopsy. Renal biopsy revealed mesangial expansion with hypercellularity, glomerular basement membrane wrinkling and thickening, and compromised glomerular capillary lumina due to neutrophil and lymphocyte infiltration. Lymphocyte-dominant inflammatory cell infiltration was observed throughout tubulointerstitial areas. Tubular epithelial cells exhibit degeneration, vacuolization, and detachment. Most of the interlobular arteries show wall thickening with inflation of lymphocyte-dominant inflammatory cells, which is consistent with small-vessel vasculitis without crescent formation (Fig. 2). The immunofluorescence study showed no immune complex such as IgG, IgM, IgA, C3, and C1q deposition. We started high-dose prednisolone (1 mg/kg/day) and cyclophosphamide (100 mg/day). However, the response was poor even after 12 weeks of treatment, and leukopenia and renal function worsened with re-elevation of anti-MPO antibodies titers (33 U/mL); therefore, we discontinued immunosuppressive therapy and decided to maintain hemodialysis (Fig. 3).

## 4. Discussion

We report a case of new-onset AAV with MPO-ANCA positivity following hantavirus infection, which presented as an atypical HFRS. Despite immunosuppressive therapy, renal function deteriorated, and hemodialysis was necessary in this case.

AAV is a group of diseases characterized by inflammation of the small blood vessels, often involving the kidney.^[4]^ The pathogenesis of AAV is not yet fully understood. However, genetic factors, bacterial infections, drugs, and environmental agents, such as silica exposure, are involved in the development of ANCA, and a few viruses, such as parvovirus B19, hepatitis B virus, and Epstein-Barr virus, have been proposed to be related to MPO-ANCA development and cause ANCA-AAV.^[13–15]^ In the era of the pandemic COVID-19, there are several reports on the association between COVID-19 infection and the development of AAV.^[2,16]^ Moreover, COVID-19 vaccination plays a role in the development of AAV.^[17]^ In this case, there was an association between a 4-fold increase in hantavirus IgG antibody titers and the positivity of the anti-MPO ANCA antibody. To our knowledge, this is the first report of AAV following hantavirus infection.

The patient showed multifocal ground glass opacity in both lung fields with fever. Broad-spectrum antibiotics were administered on suspicion of atypical pneumonia, but the clinical signs and symptoms did not improve. We administered low-dose oral prednisolone to improve the fever based on laboratory test results, including persistent eosinophilia. However, renal function deteriorated, with a 4-fold increase in the hantavirus IgG antibody titers and anti-MPO ANCA positivity. Renal biopsy confirmed AAV. Despite glucocorticoid and cyclophosphamide treatment, the renal function did not recover, and the patient required renal replacement therapy.

The hantaviruses involve endothelial cells of the renal medulla and infect other cell types in the kidney, such as tubule cells, podocytes, and glomerular endothelial cells. Therefore, AKI accompanied by hantavirus infection is thought to result primarily from endothelial damage and/or tubulointerstitial damage.^[18]^ The histopathologic finding of hantavirus infection shows acute tubulointerstitial nephritis with infiltration of inflammatory cells and immune complex-mediated crescentic glomerulonephritis also report.^[19]^

In this case, the typical course of HFRS, including febrile, hypotensive, and oliguric phases, was not found. The crescentic formation was not found by kidney biopsy. The progression of the disease and pathologic findings did not show the typical clinical presentation of AAV or HFRS in this case. However, hantavirus infection has diverse severity, and sometimes 5 phases of severe HFRS are absent, with less severity.^[19]^ We performed a serial serologic test and found a >4-fold increase of anti-hantavirus IgG, which can confirm hantavirus infection. Although crescent was absent on kidney biopsy, inflammation of small arteries was found (Fig. S1, Supplemental Digital Content, https://links.lww.com/MD/P167). Glomerular capillaries were compromised due to infiltration of neutrophils, and swollen endothelial cells were found. Inflammatory cells infiltrated most of the tubules. Berden et al^[20]^ reported that in their histopathologic classification for ANCA-associated glomerulonephritis, focal class is defined as over 50% of normal glomeruli, which shows subtle signs of ischemia: slight collapse of the tuft, focal splitting of Bowman capsule, or focal wrinkling of the GBM. They also recommend that the importance of tubulointerstitial and vascular lesions have prognostic value in ANCA-AAV. Our case shows that mesangial expansion with hypercellularity, glomerular basement membrane wrinkling and thickening, and compromised glomerular capillary lumina due to neutrophil and lymphocyte infiltration, suggesting subtle changes of ischemia without crescent formation. In addition, most of the interlobular arteries show wall thickening with inflation of lymphocyte-dominant inflammatory cells, which is consistent with small-vessel vasculitis without crescent formation. In the immunofluorescence study, there was no immunoglobulin or complement deposition. Kidney biopsy revealed pauci-immune glomerulonephritis with tubulointerstitial nephritis, which is an atypical pathology of AAV.^[21,22]^

HFRS managements are usually supportive care with correcting fluid and electrolyte balance, maintaining blood pressure, and monitoring renal function; no specific effective antiviral treatment is available.^[23]^ Some cases are required for renal replacement therapy and vasoactive drugs in patients with hypotension and shock.^[23]^

The relationship between infections and vasculitis is complex.^[24,25]^ A vasculitis is mediated by autoimmune mechanisms, infections are suspected to act as potential triggers.^[24]^ Infections can trigger vasculitis through various mechanisms, including molecular mimicry, autoantigen complementarity, or overexpression of Toll-like receptors^[6,24]^ These mechanisms can induce an immune response owing to similarities in epitopes or the inflammatory effects of infection.^[24]^ In the development of AAV, loss of T cell tolerance to neutrophils is crucial for autoantibody production.^[26]^ CD8 + T cell activation and increase of proinflammatory cytokines are likely responsible for the symptoms of hantavirus infection.^[27]^ The precise mechanism underlying the infectious trigger of AAV in ill-defined, proinflammatory stimuli may induce loss of tolerance and increase expression of cell surface MPO.^[26,28]^ Although we were not able to perform in vivo or in vitro experiments, hantavirus infection could lead to anti-MPO antibody formation by loss of T cell tolerance.

Although hantavirus has not been reported to cause vasculitis, this case suggests the need for vigilance in differentiating small-vessel vasculitis in patients presenting with AKI following viral infections. Further studies are warranted to better understand the potential of Hantavirus and other viral infections to trigger AAV.

This report describes a case of AAV with MPO antibody positivity following a Hantavirus infection. A patient’s progression from atypical pneumonia to rapidly progressive glomerulonephritis underscores the need for careful consideration of AAV in patients with AKI after viral infections. We suggest differentiating between vasculitis and other conditions in similar clinical scenarios to ensure timely and appropriate treatment.

## Author contributions

**Conceptualization:** Hyeong Wan Kim, Kyoung Min Kim, Kyung Pyo Kang.

**Data curation:** Seunghyeok Choi, Hyeong Wan Kim, Kyung Pyo Kang.

**Formal analysis:** Seunghyeok Choi, Hyeong Wan Kim, Kyoung Min Kim, Kyung Pyo Kang.

**Investigation:** Seunghyeok Choi, Hyeong Wan Kim, Kyung Pyo Kang.

**Writing – original draft:** Seunghyeok Choi, Kyung Pyo Kang.

**Writing – review & editing:** Seunghyeok Choi, Kyung Pyo Kang.

## Supplementary Material


