# Supplementary material for: ANCA-associated vasculitis after hantavirus infection: A case report
Source: Medicine (Baltimore). 2025 Jun 13;104(24):e42821. doi: 10.1097/MD.0000000000042821 (PMC12173335; doi:10.1097/MD.0000000000042821)
Supplement: Supplementary file 1 [file medi-104-e42821-s001.pptx]

## Slide 1
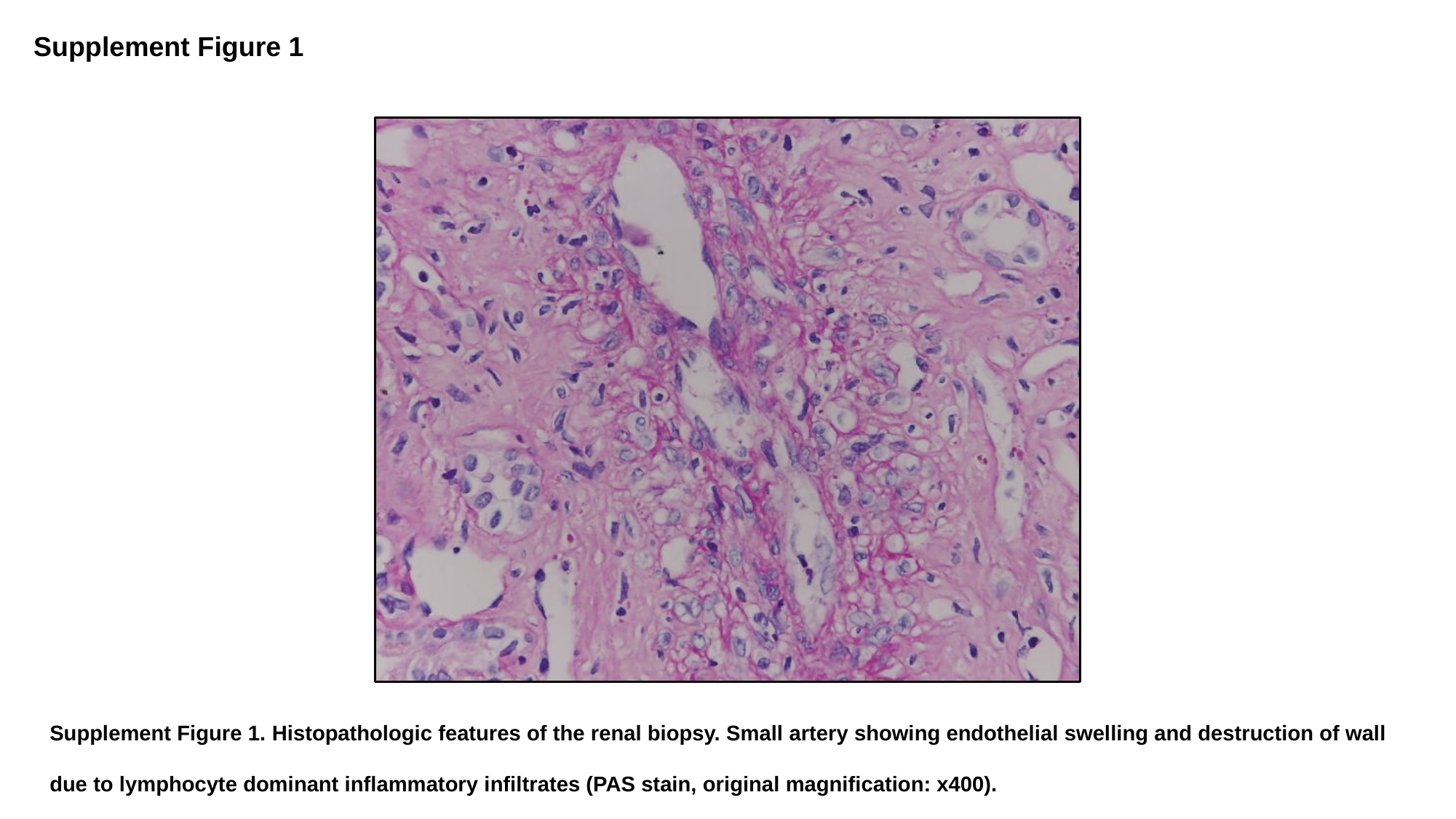

Supplement Figure 1
Supplement Figure 1. Histopathologic features of the renal biopsy. Small artery showing endothelial swelling and destruction of wall due to lymphocyte dominant inflammatory infiltrates (PAS stain, original magnification: x400).
